# Genomic Amplification and Functional Dependency of the Gamma Actin Gene *ACTG1* in Uterine Cancer

**DOI:** 10.3390/ijms21228690

**Published:** 2020-11-18

**Authors:** Camden Richter, David Mayhew, Jonathan P. Rennhack, Jonathan So, Elizabeth H. Stover, Justin H. Hwang, Danuta Szczesna-Cordary

**Affiliations:** 1 Dana-Farber Cancer Institute, Harvard Medical School, Broad Institute of MIT and Harvard, Boston, MA 02215, USA; carich21@g.holycross.edu (C.R.); DavidL_Mayhew@dfci.harvard.edu (D.M.); JonathanP_Rennhack@dfci.harvard.edu (J.P.R.); jonathan_so@dfci.harvard.edu (J.S.); Elizabeth_Stover@dfci.harvard.edu (E.H.S.); 2 Department of Radiation Oncology, Tufts Medical Center, Boston, MA 02111, USA; 3 Department of Medicine, University of Minnesota-Twin Cities, Minneapolis, MN 55455, USA; 4 Masonic Cancer Center, University of Minnesota-Twin Cities, Minneapolis, MN 55414, USA; 5 Department of Molecular and Cellular Pharmacology, University of Miami, Miller School of Medicine, Miami, FL 33136, USA; 6 Sylvester Comprehensive Cancer Center, Miller School of Medicine, Miami, FL 33136, USA

**Keywords:** *ACTG1*, cytoskeleton genes, gene dysregulation in cancer, uterine cancer

## Abstract

Sarcomere and cytoskeleton genes, or actomyosin genes, regulate cell biology including mechanical stress, cell motility, and cell division. While actomyosin genes are recurrently dysregulated in cancers, their oncogenic roles have not been examined in a lineage-specific fashion. In this report, we investigated dysregulation of nine sarcomeric and cytoskeletal genes across 20 cancer lineages. We found that uterine cancers harbored the highest frequencies of amplification and overexpression of the gamma actin gene, *ACTG1*. Each of the four subtypes of uterine cancers, mixed endometrial carcinomas, serous carcinomas, endometroid carcinomas, and carcinosarcomas harbored between 5~20% of *ACTG1* gene amplification or overexpression. Clinically, patients with *ACTG1* gains had a poor prognosis. *ACTG1* gains showed transcriptional patterns that reflect activation of oncogenic signals, repressed response to innate immunity, or immunotherapy. Functionally, the CRISPR-CAS9 gene deletion of *ACTG1* had the most robust and consistent effects in uterine cancer cells relative to 20 other lineages. Overall, we propose that *ACTG1* regulates the fitness of uterine cancer cells by modulating cell-intrinsic properties and the tumor microenvironment. In summary, the *ACTG1* functions relative to other actomyosin genes support the notion that it is a potential biomarker and a target gene in uterine cancer precision therapies.

## 1. Introduction

Actomyosin genes regulate a variety of intracellular functions, including cell migration and adhesion, intracellular transport, and signal transduction. While dysregulation of actomyosin genes is associated with diseases including cardiomyopathies [1,2,3,4,5], tumor-associated functions of actomyosin genes have been demonstrated in cancer models including colorectal, prostate, breast, ovarian, melanoma, pancreatic, and others [6,7,8]. In addition, recent studies indicate some actomyosin genes are potential target genes in metastatic cancers [9]. Functionally, mechanotransduction through actomyosin protein functions are key features of highly proliferative tumor cells, and their regulatory signaling pathways, such as RAS or G-protein coupled signaling, are indeed pro-tumorigenic [10,11,12]. In addition to modulating tensile stress, the actomyosin proteins are also signaling effectors in cell division, epithelial to mesenchymal transition (EMT), and cell motility [13,14]. While these intracellular functions are key to the maintenance of individual tumor cells, actomyosin proteins also dynamically regulate interactions with neighboring cells, the extracellular matrix, or additional factors in the tumor microenvironment [10,11,12]. Although these protein functions are well documented in the context of cancer biology, it is less apparent which cancer lineages have the greatest tendencies of dysregulating actomyosin protein functions and how clinical endpoints such as tumor progression or overall survival are impacted by these alterations in patients.

Our inquiry of actomyosin gene functions in cancers coincides with the expansive efforts of cancer gene profiling through genomics [15,16] or functional screens [17,18,19]. In public portals [15,16], we can now compare the relative dysregulation of each gene in cancer tissue in close to three hundred studies including twenty cancer lineages with more than forty-five thousand samples. In parallel to these efforts, gene function in cancers can also be broadly evaluated at a genome-scale, based on CRISPR-CAS9 gene deletion screens in over seven hundred cancer cell lines in over twenty cancer lineages [17,18,19]. Altogether, these resources allow us to nominate the cancer lineages with tendencies to dysregulate the function of actomyosin genes.

In this report, we focus our investigation on the major myosin and actin genes, two classes of proteins whose perpetual interaction underlies vital functions of the human body. Given the breadth of knowledge regarding their protein structures and mechanistic interactions in the contexts of normal tissue, identifying the recurrent cancer-specific gene alterations provides a foundation towards understanding the essential biology and mechanisms by which they promote cancer malignancy.

## 2. Results

### 2.1. Actomyosin Genes Are Dysregulated in Cancer

**Figure 1:**
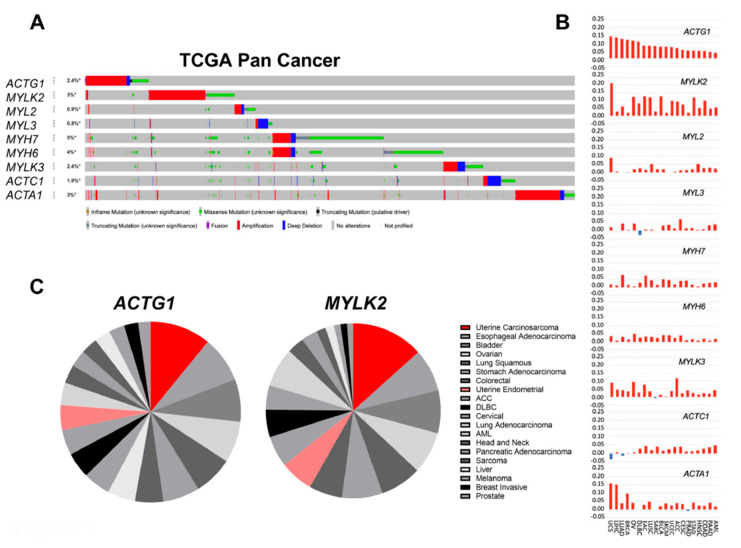
Uterine cancers harbor robust dysregulation in *ACTG1* and *MYLK2*. (**A**) An OncoPrint that represents the genomic dysregulation status of muscle and structural genes in 32 TCGA Pan-Cancer studies (n = 10,967). Each row is ordered by the patient ID and each colored vertical lines represent the observance of genomic alteration in one patient. The percentages on the left of each row represent the total alteration frequencies among all patient samples. The color of the vertical lines represents genomic amplifications (red), deletions (blue), and mutations (green). All genes were genomically dysregulated in TCGA studies and confirmed in Pan-Cancer studies. (**B**) Net Gain Score of gene expression and copy-number variation is shown (y-axis) in 20 cancer lineages in TCGA Pan-Cancer studies. UCS = Uterine carcinosarcoma; LIHC = Liver hepatocellular carcinoma; LUAD = Lung adenocarcinoma; BRCA = Breast invasive carcinoma; OV = Ovarian serous cystadenocarcinoma; DLBC = Lymphoid Neoplasm Diffuse Large B-cell Lymphoma; EAC = Esophageal adenocarcinoma; LUSC = Lung squamous cell carcinoma; SARC = Sarcoma; BLCA = Bladder urothelial carcinoma; SKCM = Skin Cutaneous Melanoma; UCEC = Uterine corpus endometrial carcinoma; ACC = Adrenocortical carcinoma; CESC = Cervical squamous cell carcinoma and endocervical adenocarcinoma; PRAD = Prostate adenocarcinoma; STAD = Stomach adenocarcinoma; HNSC = Head and neck squamous cell carcinoma; COAD = Colon adenocarcinoma; PAAD = Pancreatic adenocarcinoma; AML = Acute Myeloid Leukemia. (**C**) A pie chart of *ACTG1* and *MYLK2* gains (amplifications and overexpression) across the 20 cancer lineages. Uterine carcinosarcoma is highlighted in red and uterine endometrial is highlighted in pink.

**Figure 2:**
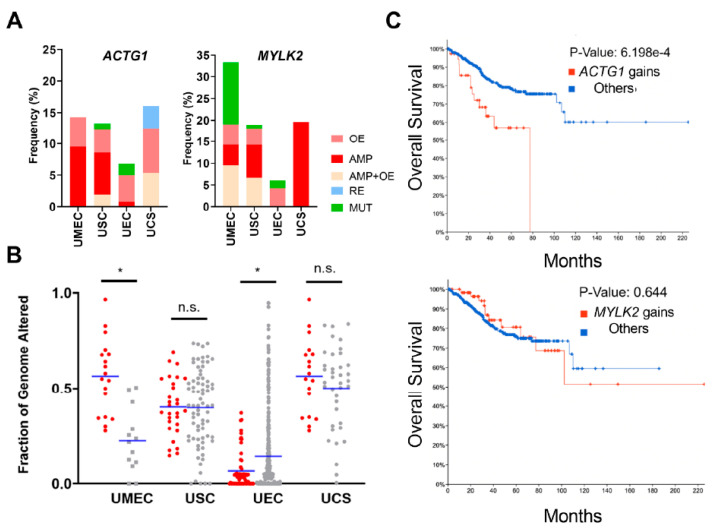
*ACTG1* gains are consistently observed across uterine cancer subtypes and predict worse prognosis. (**A**) *ACTG1* and *MYLK2* genomic alterations in uterine cancers; UMEC = Uterine mixed endometrial carcinoma; USC = Uterine serous carcinoma; UEC = Uterine endometrioid carcinoma; UCS = Uterine carcinosarcoma. OE = overexpression; AMP = amplification; AMP + OE = % amplification + % overexpression; RE = reduced expression; MUT = mutation. Overexpression is indicated by high levels of mRNA while reduced expression is indicated by low levels of mRNA. (**B**) The fraction of the genome (FGA) that is altered in UMEC, USC, UEC and UCS. Red dots indicate *ACTG1* and *MYLK2* dysregulated tumors, grey dots indicate all other genes in that cancer subtype. (**C**) Kaplan-Meir curves for *ACTG1* and *MYLK2* in uterine corpus endometrial carcinoma. The altered group represents overall gain, including amplification and overexpression. A *p*-value of less than 0.05 is considered a statistically significant difference, thus, *ACTG1* predicts poor prognosis in uterine cancer.

To identify mutation patterns, we examined the alteration frequency in different lineages for nine muscle and cytoskeletal genes (Supplemental Figure S1) as well as determined the alteration rates for putative cancer regulatory genes (Supplemental Figure S2). We examined ~46 K Pan-Cancer samples, including those from The Cancer Genome Atlas (TCGA) as well as other studies. For TCGA studies (number of samples, n = 10,967), we specifically examined the gene level dysregulation which consisted of amplifications, deletions, mutations, mRNA up-regulation, or mRNA down-regulation. In TCGA studies, we found all of the nine selected genes were genomically and transcriptionally dysregulated in 20 cancer lineages (Figure 1A,B). We confirmed trends of genomic alterations in a larger cohort of 176 Pan-Cancer studies (n = 46,697, Supplemental Figure S1). All genes were dysregulated at the gene level including *MYH7* and *MYH6* that encode for the β- and α-myosin heavy chain (MHC), respectively. They showed the greatest frequency of modification by missense mutations (Figure 1A, Supplemental Figure S3). *MYH6* and *MYH7*, both on 14q11.2, were expectedly co-amplified and deleted in the same tumor samples (Supplemental Figure S3A) with significant missense and truncation mutations distributed throughout both genes (Supplemental Figure S3B). The 14q11.2 locus has been previously shown to contain the driver alteration of a prosurvival member of the BCL2 family *BCL2L2* gene [20]. Our results demonstrate that *MYH6* or *MYH7* amplification events are seldom independent of *BCL2L2* (Supplemental Figure S4A), suggesting these are passenger events. While skeletal α-actin gene, *ACTA1*, was recurrently amplified or overexpressed, its amplifications and deletions were co-regulated with *MDM4* at chromosome 1q42.13 across cancers. Given the oncogenic functions of *MDM4*, and that few *ATCA1* amplification events were independent of *MDM4* (Supplemental Figure S4B), it was unclear if *ACTA1* had an independent oncogenic role based on genomic analysis. Among the actomyosin genes, *ACTG1* (cytoplasmic γ-actin) and *MYLK2* (skeletal myosin light chain kinase) were consistently amplified or overexpressed across cancers (Figure 1A,B). Notably, *MYLK2* and *ACTG1* reside on separate chromosomes (20q11 and 17q25, respectively), indicating that separate genetic events lead to their dysregulation. Uterine carcinosarcomas (UCS) were the lineage with the greatest rates of genomic or transcript upregulation of *MYLK2* and *ACTG1*, with Net Gain Scores of 0.21 and 0.14 respectively (Figure 1B and Figure 2A). Although of distinct histology and pathogenesis, uterine corpus endometrial carcinomas (UCEC), which includes multiple subtypes, the majority being uterine endometrioid carcinoma (UEC) as well as uterine serous carcinoma (USC) and uterine mixed endometrial carcinoma (UMEC), also upregulated *ACTG1* and *MYLK2* (Figure 1B,C). Overall, our analysis predicted potential regulatory roles for *MYLK2* and *ACTG1* in uterine cancers.

### 2.2. Uterine Cancer Subtypes Harbor Significant Gains of ACTG1 with Poor Prognosis in Patients

Given that *ACTG1* and *MYLK2* showed the greatest alteration frequency in uterine cancer, we sought to explore them further within uterine cancer subtypes. *ACTG1* and *MYLK2* were gained (amplified and/or overexpressed) at 5~14% and 5~20%, respectively, in uterine mixed endometrial carcinoma (UMEC), uterine serous carcinoma (USC), uterine endometrioid carcinoma (UEC) and uterine carcinosarcoma (UCS) (Figure 2A). To determine potential mechanisms of *ACTG1* and *MYLK2* gains, we also examined genomic instability through measurements of fraction of the genome altered (FGA) in uterine cancers with and without *ACTG1* and *MYLK2* gains. USC and UCS did not have a statistically significant difference between the two groups, indicating that the *ACTG1* and *MYLK2* dysregulation in those tumors was not driven by genomic instability (Figure 2B). However, in UEC, these gains were associated with statistically significantly lower rates of FGA, whereas in UMEC, we observed higher FGA. Overall, we found no consistent relationship between *ACTG1* and *MYLK2* dysregulation and FGA, indicating that these events were likely not reflective of overall genomic instability.

Given that *ACTG1* and *MYLK2* amplifications or overexpression were consistently observed in uterine cancers, we sought to analyze their potential as uterine cancer biomarkers. We found that patients with *ACTG1* gains in the UCEC cohort, relative to those without, had a worse prognosis, while patients with *MYLK2* gains did not. Based on Kaplan-Meier curves, *ACTG1* gains predicted significantly shorter overall survival (*p*-value = 6.198 × 10^−4^, Figure 2C). We also examined if nearby genes in the same genomic loci as *ACTG1* also predicted poor survival and found that alterations in *NPLOC4* (located at 17q25.3) were not associated with poor survival (Supplemental Figure S5).

### 2.3. Clinical, Histological and Molecular Features of ACTG1 Gains

**Figure 3:**
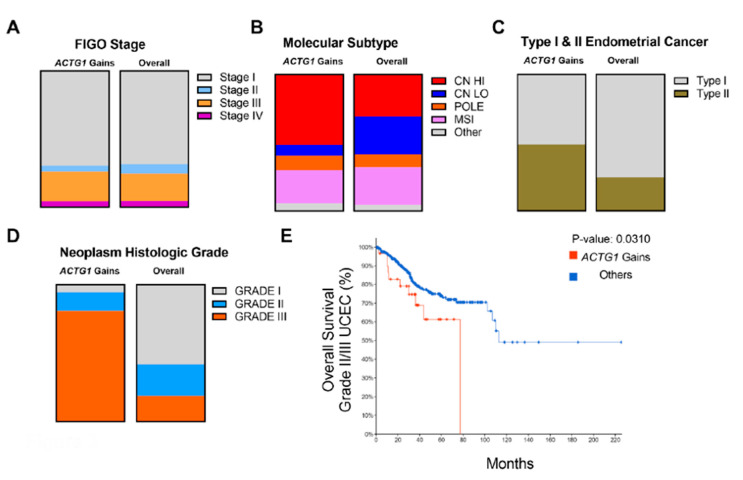
Distribution of *ACTG1* gains in uterine corpus endometrial carcinoma (UCEC). (**A**) Stages of UCECs with *ACTG1* gains (n = 23) or overall (n = 355) are shown as a proportion based on the FIGO staging system. (**B**) Relative to overall (n = 529), the proportion of *ACTG1* gains (n = 37) are shown based on molecular subtypes in UCEC. (**C**) The proportion of *ACTG1* (n = 37) gains relative to overall (n = 529) are shown in type I (endometroid) and type II (all non-endometrial) tumors. (**D**) Neoplasm histologic grade of UCECs are shown for tumors with *ACTG1* gains (n = 36) or overall (n = 518). (**E**) Overall survival in Grade II/III UCEC tumors is shown as a Kaplan-Meier curve for with tumors with and without *ACTG1* gains.

Given that *ACTG1* gains were associated with worse prognosis, we sought to further explore the genetic interactions of *ACTG1* based on known classifications of UCEC including FIGO Stage, histological grade, molecular subtypes, and tumor type [21,22]. We did not find a significant difference between the percentage of cases across the FIGO stage with *ACTG1* gains vs. overall cases (Figure 3A). Next, we examined the molecular subtypes associated with endometrial carcinomas. TCGA classified UCECs into the POLE ultra-mutated, microsatellite instability (MSI), copy number low and copy number high groups [21,22]. *ACTG1* gains were enriched in the population of copy number high tumors but reduced in the copy number low tumors (Figure 3B). This is consistent with our finding that many cases harbored *ACTG1* copy number gains (Figure 2A). We also examined *ACTG1* gains with consideration of type I vs. type II endometrial cancers. We found that *ACTG1* gains were slightly enriched in type II endometrial cancers (Figure 3C). Moreover, while we further looked at *ACTG1* gains among tumors with distinct histologic grades, we found *ACTG1* gains in greater proportion in grade III neoplasms and had reduced representation in grade I neoplasms (Figure 3D). As grade 1 tumors have an exceptional prognosis, they were excluded from survival analysis to enrich for clinical events relevant to *ACTG1* gains. *ACTG1* gains remained prognostic for grade II/III tumors (Figure 3, *p* = 0.031). Together, this data suggests that while *ACTG1* gains occur more commonly in higher grade uterine cancers, they nonetheless remain prognostic for overall survival in this cohort. (Figure 3E). Overall, *ACTG1* gains were not limited to specific UCEC clinical characteristics. In addition, the enrichment of *ACTG1* gains in high-grade tumors specified a group of patients with even worse prognosis.

### 2.4. ACTG1 Gains in UCEC are Associated with Cell Cycle, PRC2 Activity, and Epithelial to Mesenchymal Transition

**Figure 4:**
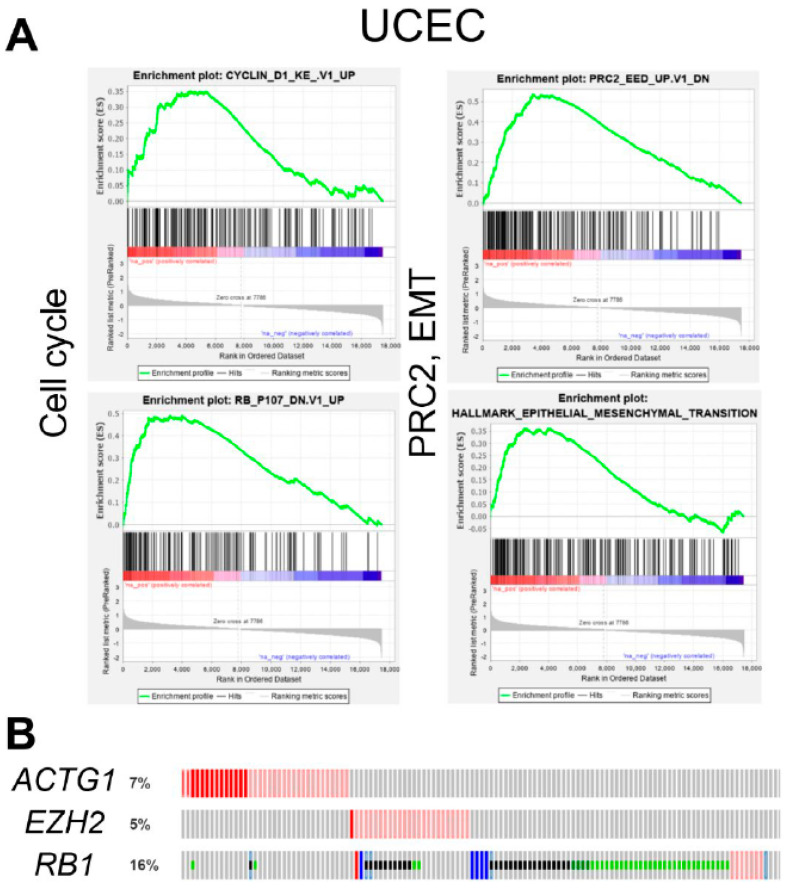
UCEC with *ACTG1* gains exhibits increased cell cycle, EMT, and PRC2 activity. (**A**) Gene Set Enrichment Analysis (GSEA) of *ACTG1* in the UCEC cohort (n = 509 samples) shows enrichment of cell cycle (cyclin and RB), epithelial to mesenchymal transition (EMT) and PRC2 activity. The peak of the graph indicates the enrichment score, the bars in the middle show where the genes related to the pathway are located in ranking (red/left = positive correlation, blue/right = negative correlation). (**B**) OncoPrint of genetic alterations in *ACTG1, EZH2*, and *RB1*. Each row is ordered by the patient ID and the colored vertical lines represent the observance of genomic alteration in one patient; red = amplification, pink = overexpression, blue = deletion, green = mutation. *ACTG1* and *EZH2* are not altered in the same patient samples.

To further understand the role of *ACTG1* in driving worse patient outcomes in UCEC, we used Gene Set Enrichment Analysis (GSEA) to examine biology based on gene expression profiles in patients with or without *ACTG1* gains. GSEA identified significantly enriched transcriptional patterns that demonstrate *ACTG1* gains are associated with the increased expression of cell cycle (cyclin D1 and RB), epithelial to mesenchymal transition (EMT), and PRC2 (a histone methyltransferase) activity gene sets in the UCEC cohort (Figure 4A). Further, while *ACTG1* gain was found to be associated with increased PRC2 activity, *EZH2*, a core member of the PRC2 complex and an oncogenic target in several cancers [23,24,25,26] was not altered in the same patient samples (Figure 4B). Analogously, increased cell cycle activity was observed in the absence of *RB1* loss, a putative mechanism of cell cycle dysregulation in uterine cancers (Figure 4B) [21,27,28]. These observations indicate *ACTG1* gains in UCEC may support key oncogenic processes without directly changing the expression of key regulatory genes in the respective signaling pathways. In addition, we found tumors with ACTG1 gains were associated with reduced *KRAS* signaling (Supplemental Table S2), however this observation was in concordance with an apparent enhancement of MEK activity (Supplemental Table S4), the key effector kinase of RAS proteins, suggesting a *KRAS*-independent mechanism of MEK1/2 activation.

### 2.5. ACTG1 Gains Correspond to Repressed Immune Signaling Response and Lymphocyte Infiltration

**Figure 5:**
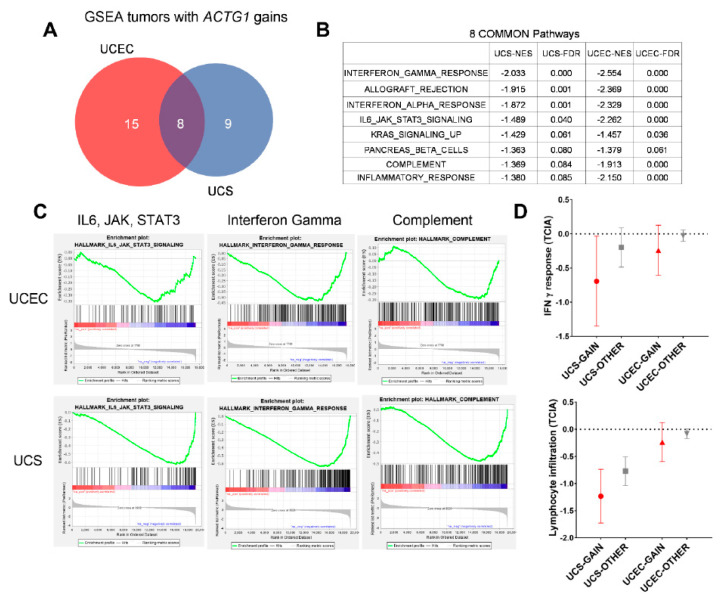
UCEC and UCS with *ACTG1* gains are associated with a reduction in an interferon gamma response (IFN-γ) response. (**A**) Venn diagram represents GSEA analysis of UCEC and UCS with *ACTG1* gains from TCGA, with the numbers representing statistically significant gene sets (FDR < 0.1) (**B**) 8 gene programs are consistently enriched in UCEC and UCS with *ACTG1* gains. The net enrichment scores (NES) and false discovery rates (FDR) are shown. (**C**) Enrichment plots of *ACTG1* in UCS and UCEC cohorts show de-enrichment in interleukin-6 (IL6) and IFN-γ. (**D**) Based on aggregating TCIA data, the IFN-γ response and lymphocyte infiltration tumors examined within the UCS and UCEC cohorts and grouped by *ACTG1* gain (red) and without (grey). The mean response is shown as well as a 95% confidence interval (CI). A *p*-value of less than 0.1 was considered significant.

To identify common biology among all uterine cancer samples with *ACTG1* gains, we performed GSEA in all the uterine tumors from the UCS and UCEC cohorts in The Cancer Immunome Atlas (TCIA) (Figure 5A). Given these are tumors with distinct histology, we found that many of the enriched pathways were expectedly distinct (Supplemental Tables S2 and S3). Interestingly, of the top-ranked gene programs based on net enrichment scores (NES) and statistical significance (FDR < 0.1), *ACTG1* gains were generally associated with tumors that exhibit differential regulation of cytokine signaling or immune regulatory responses (Figure 5B). Specifically, the patterns of enrichment indicated that *ACTG1* gains correlated with a reduction in expression of these pathways, including IL6, JAK/STAT signaling, interferon gamma and alpha response, as well as complement (Figure 5C). TCIA has previously examined 60 immune-associated phenotypes of samples within TCGA based on expression profiles [29]. Of tumors within the UCS and UCEC cohorts, we found that UCS and UCEC tumors with *ACTG1* gains indeed exhibited lower IFN-γ signaling and UCS tumors with *ACTG1* gains exhibited lower lymphocyte infiltration (IFN-γ: UCS *p*-value = 0.081, UCEC *p*-value = 0.092; Lymphocyte infiltration: UCS *p*-value = 0.073, UCEC *p*-value = 0.189) (Figure 5D). Overall, these orthogonal approaches indicate uterine tumors with *ACTG1* gains have repressed IFN-γ and lymphocyte infiltration, which suggests these tumors may exhibit differential responses towards innate immunity or even immunotherapies.

### 2.6. ACTG1 is a Dependency Gene in Uterine Cancer

**Figure 6:**
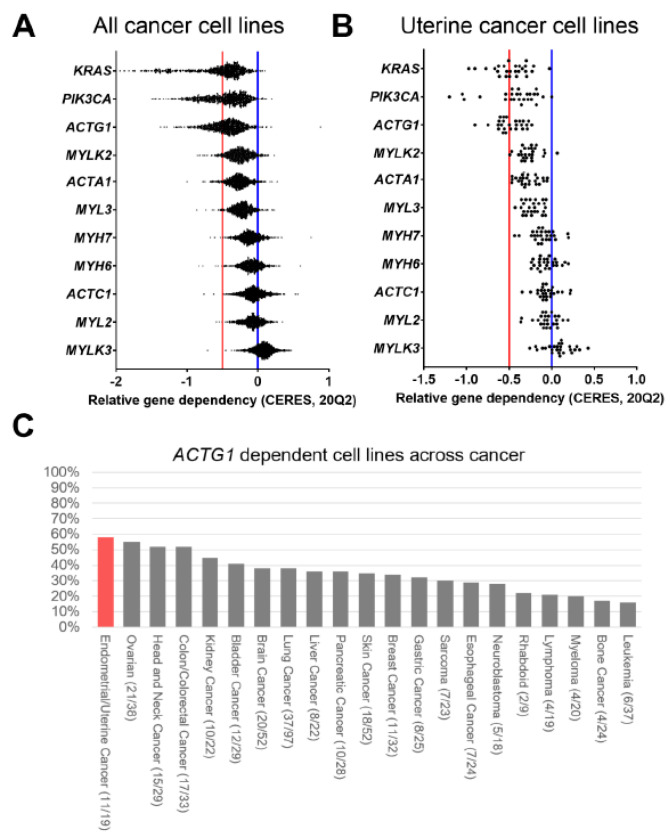
*ACTG1* is a uterine cancer dependency based on CRISPR-Cas9 depletion screens. (**A**) The relative dependency of actomyosin genes, including *ACTG1*, is shown (20Q2 release) along with the scores of the known oncogenes, *KRAS* and *PIK3CA* for all cell lines (n = 769). (**B**) The relative dependency in 19 uterine cancer cell lines is shown for actomyosin and control genes. (**C**) Cancer cell line lineages are ranked based on the percentage of cell lines that exhibited *ACTG1* dependency.

Since *ACTG1* predicted poor prognosis and was associated with pro-tumorigenic signaling pathways such as cell cycle, PRC2, and EMT, we also postulated that *ACTG1* may regulate cell-intrinsic properties required for tumor cell fitness. Among 739 cancer cell lines, we examined the potential function of all actomyosin genes based on genome-scale CRISPR-Cas9 gene depletion screens. From observing decreased viability upon gene depletion, we found *ACTG1* was a dependency gene in almost a third of the cancer cell lines (n = 254, Figure 6A). The data indicates that *ACTG1* was not a common essential gene that upon gene deletion globally perturbs cancer cell viability. This frequency of *ACTG1* dependency exceeded all the other sarcomeric and cytoskeletal genes in our set, including the recurrently amplified and overexpressed gene *MYLK2* (n = 33 and 3, respectively). This frequency was also shown to be comparable to previously known oncogenes, *KRAS* and *PIK3CA*. Among 21 cancer lineages with *ACTG1* dependent cell lines, 4 lineages had more than half the cell lines score as *ACTG1* dependent; 58% of endometrial/uterine cancer cell lines; 55% of ovarian cancer cell lines; 52% of head and neck cancer cell lines; and 52% of colon/colorectal cancer cell lines (Figure 6C). Further, *ACTG1* was the only actomyosin gene that regulates the fitness of uterine cancer cell lines (Figure 6B, Supplemental Table S5). While our observations confirm a functional role of *ACTG1* in uterine cancers, the results also implicate *ACTG1* expression is essential for the fitness of several other cancer lineages, including that of ovarian and lung.

## 3. Discussion

**Figure 7:**
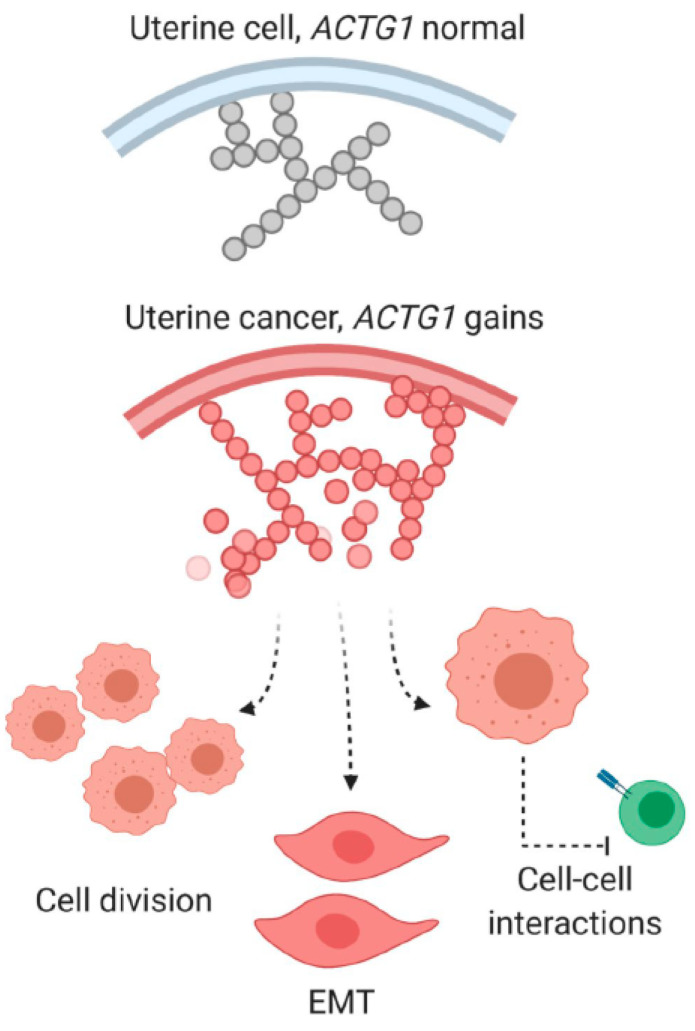
*ACTG1* function and consequences of its gains are depicted in uterine cancers subtypes.

Gene regulation in oncogenesis is a central topic in modern molecular biology, and elucidating the underlying mechanisms is critical to understanding the biology of cancer and is crucial for developing target-specific diagnostic and therapeutic measures. Our inquiry of actomyosin genes’ functions in cancers coincides with the expansive efforts of cancer gene profiling through genomics [15,16] or functional screens [17,18,19]. Examination of nine sarcomeric and cytoskeletal genes across 20 cancer types showed that uterine cancers harbored the greatest rates of amplification and overexpression of the γ-actin gene, *ACTG1*. Two of the three actin genes that we analyzed in this report, *ACTA1* (α-skeletal) and *ACTC1* (α-cardiac) are primarily found in muscle cells, whereas *ACTG1* is ubiquitously and highly expressed in non-muscle cells [30]. In this study, we found *ACTG1* was associated with several dysregulated transcriptional profiles and features in uterine cancers (Figure 7).

### 3.1. ACTG1 is an Amplified Gene That Predicts Poor Prognosis in Uterine Cancer Subtypes

Based on previous clinical genomic interrogations, pathways including P53, RAS, and PI3 Kinase are critical oncogenic regulators of uterine cancers [21] and the aggressive UCS subtype [27,28]. Actomyosin genes are effectors of oncogenic pathways that regulate biology essential for the progression, metastatic potential, and interaction of tumor cells [7,10,11,21]. Our examination across multiple cancers identified that uterine cancers harbored the most frequent genomic and transcriptional up-regulation of the γ-actin gene, *ACTG1*, and the myosin light chain kinase gene, *MYLK2*. Up to 20% of samples with gains were observed across each subtype including UMEC, USC, UEC, and UCS with limited mutations or deletions. Clinically, *ACTG1* gains predicted poor prognosis in UCECs, which was also supported by enrichments of tumor-promoting pathways in the same samples. Tumors with *MYLK2* gains did not show a worse prognosis. Besides, no other actomyosin genes we examined exhibited as robust rates of gene dysregulation. While these observations may dismiss the prognosis utilities of *MYLK2*, as well as other recurrently perturbed actomyosin genes, e.g., *MYH6 and MYH7*, the specific functions of these genes still require a detailed functional interrogation in uterine as well as other cancer models.

### 3.2. Intrinsic Functions of ACTG1 in Uterine Cancer Cells

Of the UCEC patients with *ACTG1* gains, we also identified the enrichment of several oncogenic signatures, including increases in EMT, PRC2, and cell cycle activity (Figure 4A and Supplemental Table S3). Of these pathways, genomic and transcriptional interrogation has implicated EMT as a critical modifier of biology in uterine cancers [21,27,28]. At the cellular level, EMT encompasses alterations in tensile strength, mechanotransduction, and intracellular cytoskeletal organization, enhancing a cell’s migratory and invasive properties [10,11,12]. While one may surmise that EMT, as well as PRC2, and cell cycle functions are each critical mechanisms required for the optimal survival of uterine cancer cell lines, it remains unclear if *ACTG1* deletion directly impacts these pathways in the ~50% of uterine cancer cell lines that we deemed *ACTG1* dependent (Figure 6). Further, the association of *ACTG1* gains with increased PRC2 activity in the absence of *EZH2* alteration and with cell cycle alterations in the absence of *RB1* loss indicates the support of oncogenic processes without altering key regulatory genes. *EZH2* plays a critical role in cell proliferation and its overexpression is associated with cancer metastasis and poor prognosis [23,24,25,26]. It is also currently a proposed target in cancers such as B-cell lymphomas (Clinical Trial ID: NCT01897571, NCT02220842), adult sarcomas (NCT03874455) and uterine cancers (NCT03348631). Understanding how *ACTG1* may affect PRC2 activity in the absence of these regulatory genes potentially elucidates novel mechanisms of PRC2 functions in uterine cancer. Lastly, activated RAS signaling is considered a critical oncogenic pathway in UCEC and UCS [21,27,28], however we found the subset of tumors with *ACTG1* gains were associated with relatively reduced KRAS signaling (Supplemental Table S3). Simultaneously, we observed patterns that would indicate enhanced activity of MEK, a key effector kinase of RAS proteins. In non-uterine cancer models, RAS targets MEK signaling to uncouple Rho from the assembly of stress fibers, thus disrupting the actin cytoskeleton, reducing cell adhesion and enhancing cell motility [31]. Our observation in which *ACTG1* gains were associated with reduced KRAS signaling yet enhanced MEK activity may indicate that *ACTG1* gains interact with MEK through other RAS family members [32] or through non-canonical, RAS-independent mechanisms [33,34]. Overall, our results imply a need to develop functional uterine cancer models to study how *ACTG1* protein levels modulate key biology and signaling pathways in 2D or even 3D models.

### 3.3. Immune Regulatory Functions of Uterine Cancers with ACTG1 Gains

Subsets of uterine cancers exhibit high tumor mutational burdens (TMB) [27,35], microsatellite instability (MSI) [36], as well as gene expression profiles associated with improved response to immunotherapies [36,37,38]. These clinically translatable observations in uterine cancer samples are now rationally supported by stage III clinical trials that examine the benefits of immune therapies for uterine cancer patients (Clinical Trial ID: NCT03884101, NCT03517449) [39]. While the outcomes of these studies are pending, understanding biomarkers of response or lack thereof will become essential for patients receiving these treatments. Enriched in UCEC with *ACTG1* gains, EMT regulates tumor evasion of innate immunity [40,41,42] or immune therapies [42,43]. In concordance with this, we found *ACTG1* gains in UCEC were associated with multiple immunosuppressive phenotypes including decreases in signaling of IL6, JAK/STAT, Interferon alpha and gamma, and complement (Figure 5), as well as a projected reduction in lymphocyte infiltration by TCIA [29], suggesting enrichments in EMT could be associated with such immunosuppressive phenotypes. However, whether *ACTG1* is indeed a marker of uterine cancer response to immune therapies can only be determined once the genomics and transcriptomics of resistant patients are fully characterized from these clinical studies. In the preclinical setting, this also requires the perturbation of *ACTG1* in models in which one can evaluate uterine cancer cell interaction with immune cells, such as immune-competent tumor-forming mouse models or co-culture models in vitro.

### 3.4. Clinical Utility of ACTG1 Gains in Uterine Cancer Patients

The rising incidence of uterine cancers in the population and the poor prognosis with advanced disease (with minimal improvement in mortality rates over the past 30 years) will lead to a projected 12,590 deaths from uterine cancers in 2020, per National Cancer Institute (NCI) Surveillance Epidemiology, and End Results Program (SEER). New therapeutic targets are needed for this disease. While mechanisms that target actin cytoskeleton genes are proposed forms of cancer therapeutics in other studies [44,45] our findings indicate precision strategies against *ACTG1* could yield benefits for a subset of uterine cancer patients with high-grade tumors. Overall, further elucidating the tumorigenic role of *ACTG1* gains in uterine cancers potentially identifies critical biology underlying the pathogenesis of uterine cancers. The understanding may translate to impact precision therapeutic strategies that would benefit a subset of uterine cancer patients with a worse prognosis.

## 4. Methods

### 4.1. Gene Dysregulation Status Analysis

In this report, we focused our investigation on major sarcomeric and cytoskeleton genes: *MYH7*, *MYH6*, *MYL2*, *MYL3*, *MYLK2*, *MYLK3*, *ACTC1, ACTA1 and ACTG* encoding for β-MHC, α-MHC, and cardiac RLC, cardiac ELC, skeletal MLCK, cardiac MLCK, as well as cardiac α-actin, skeletal α-actin, and cytoplasmic γ-actin, respectively (Supplemental Table S1).

Copy-number variation, expression, and mutational data were collected from cBioPortal, a web platform for cancer genomics consisting of large-scale cancer genomics data sets [15,16]. The Cancer Genome Atlas supported by the NIH-NCI and NHGRI consortium was used. For genes of interest, expression and copy-number variation data were first collected from TCGA Pan-Cancer Atlas datasets prior to analysis. Gene dysregulation was also analogously examined in 176 Pan-Cancer studies. OncoPrint, by which multiple genomic changes can be presented in the form of a heat map, was used to visualize alteration trends in cohorts of cancer patients. After extracting data from sets with expression data, alteration frequencies were calculated for mRNA expression (high/low), amplification, and deletion. We then calculated the Net Gain Scores by adding mRNA high and amplification together and subtracting mRNA low and deletion. Net Gain Scores were graphed for each gene of interest across the top twenty cancers and alteration frequencies were graphed for uterine cancer subtypes.

### 4.2. Survival Curves

To analyze the potential of genes as survival biomarkers, we examined de-identified clinical data from cBioPortal, the cancer genomics portal containing large-scale cancer genomics data sets that included overall and disease-free survival data. Log-rank tests were performed to determine the statistical significance of differences between the two groups and *p*-values of less than 0.05 indicated statistically significant differences.

### 4.3. Dependency Analysis

For genes of interest, dependency scores were collected from the Depmap Portal [17,18,19] (20Q2 release). This is a consortium of genome-scale CRISPR screens that test the function of all genes in pools across 739 cancer cell lines in proliferation assays. Gene function, or relative viability, is represented as CERES dependency scores in the 20Q2 data set [46]. Negative scores indicate that when the target gene is knocked out, the cancer cell line lost viability. We considered −0.5 as dependent in our analysis. When analyzing the fraction of *ACTG1* dependent cell lines across cancers, we ranked relative dependency of cancer lineages upon setting thresholds at two standard deviations below the mean (n > 16 cell lines).

### 4.4. Gene Set Enrichment Analysis (GSEA) of Uterine Cancers

We utilized GSEA [47] to interpret gene expression to nominate transcriptional programs that are associated with uterine cancers with *ACTG1* gains. Expression data from uterine carcinosarcoma and uterine corpus endometrial carcinoma TCGA Pan-Cancer studies were classified based on *ACTG1* gain status using data from cBioPortal. The log ratios were ranked and entered into GSEA. Hallmark gene sets and also oncogenic signaling pathways that represent well defined biological states via coordinated patterns of gene expression were examined. Pathways with a false discovery rate (FDR) of less than 0.1 were considered significant in this analysis. Pathways were ranked by their net enrichment score (NES).

### 4.5. TCIA Analysis

Tumor profiles in the UCS and UCEC cohorts were examined based on The Cancer Immunome Atlas (TCIA) [29]. 60 pre-nominated categories were compared with interferon gamma signaling and lymphocyte infiltration demonstrating consistent differences when considering *ACTG1* gains.

## Supplementary Materials

Supplementary materials can be found at https://www.mdpi.com/1422-0067/21/22/8690/s1. Figure S1. Genomic alterations of actomyosin genes in Pan-Cancer analysis. Figure S2. Genomic alterations of putative tumor promoting and suppressing genes. Figure S3. Genomic alterations of *MYH6* and *MYH7*. Figure S4. Genomic amplifications of key actomyosin genes in the same locus. Figure S5. Overall survival of patients with *NPLOC4* in uterine cancer patients. Table S1. Nine actomyosin genes and their genomic locations. Table S2. Hallmark pathways enriched in UCEC. Table S3. Hallmark pathways enriched in USC. Table S4. Oncogenic signaling pathways enriched in USC and UCEC. Table S5. Details of uterine cancer cell lines.

## Abbreviations

*ACTA1*	Skeletal α-actin
*ACTC1*	Cardiac α-actin
*ACTG1*	Cytoplasmic γ-actin
*MYH6*	α-Myosin Heavy Chain (MHC)
*MYH7*	β-MHC
*MYL2*	Myosin Regulatory Light Chain (RLC)
*MYL3*	Myosin Essential Light Chain (ELC)
*MYLK2*	Skeletal Myosin Light Chain Kinase (MLCK)
*MYLK3*	Cardiac MLCK
TCGA	The Cancer Genome Atlas
TCIA	The Cancer Immunome Atlas
GSEA	Gene Set Enrichment Analysis
UCEC	Uterine Corpus Endometrial Carcinoma
UCS	Uterine Carcinosarcoma
UEC	Uterine Endometroid Carcinoma
UMEC	Uterine Mixed Endometrial Carcinoma
USC	Uterine Serous Carcinoma
