# Supplementary material for: Genomic Amplification and Functional Dependency of the Gamma Actin Gene ACTG1 in Uterine Cancer
Source: Int J Mol Sci. 2020 Nov 18;21(22):8690. doi: 10.3390/ijms21228690 (PMC7698702; doi:10.3390/ijms21228690)
Supplement: Supplementary file 1 [file ijms-21-08690-s001.pdf]

Pan Cancer

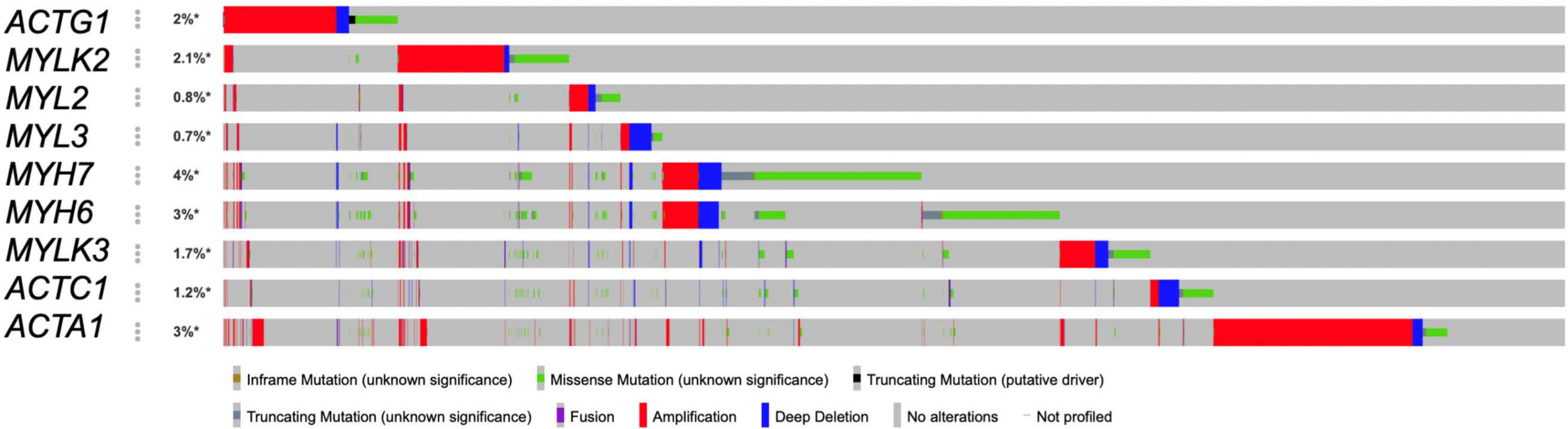

Supplemental Figure 1. Trends of genomic alterations are confirmed in a greater cohort of 176 Pan Cancer studies (n=46697).

Supplemental Figure 1

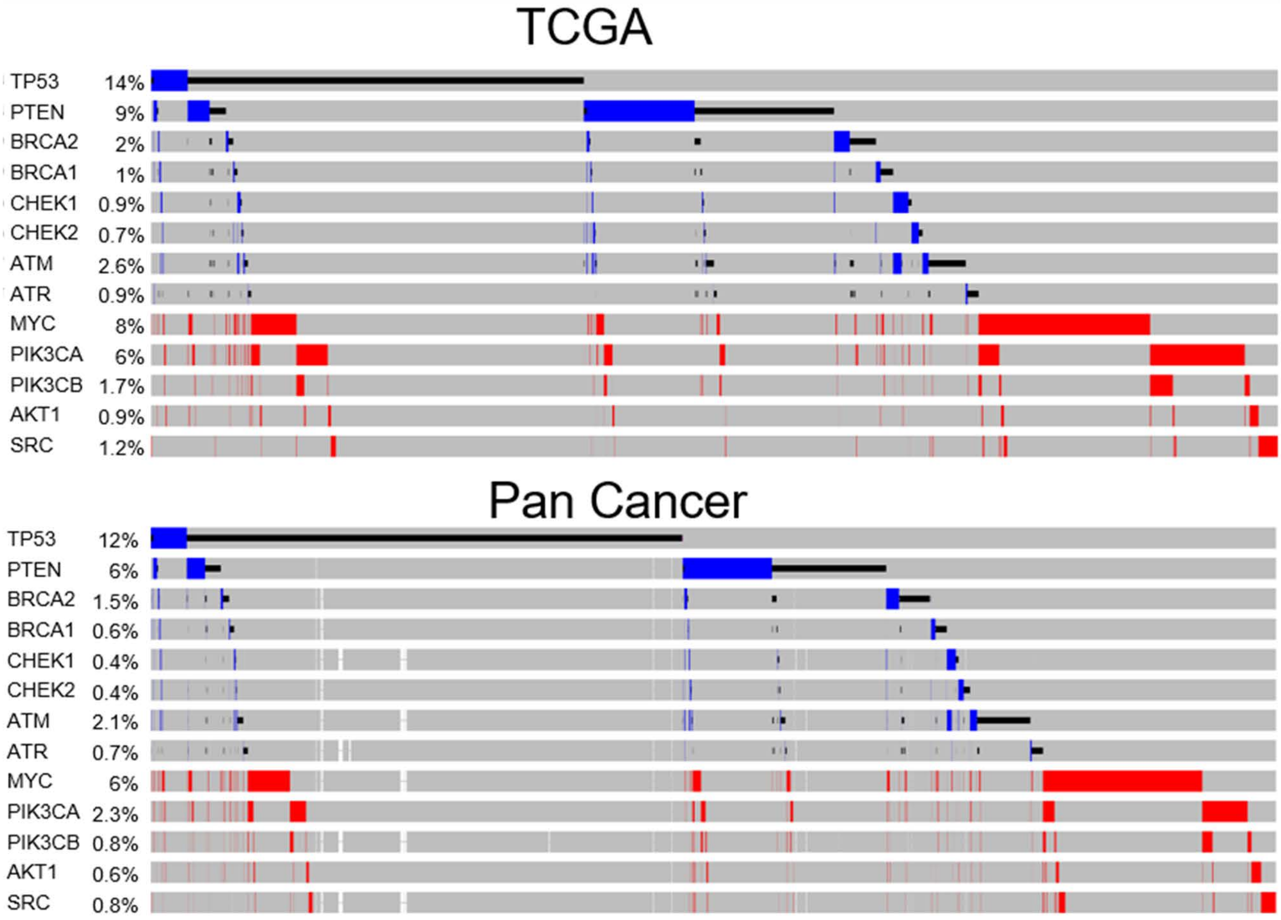

Supplemental Figure 2. Genomic dysregulation of tumor suppressor or oncogenes in cancers. Deletions (blue) and truncation mutations (black) for tumor suppressor genes and amplifications (red) and oncogenes are shown in (A) 32 TCGA Pan Cancer studies (n=10967) as well as (B) 176 Pan Cancer studies (n=46697).

Supplemental Figure 2

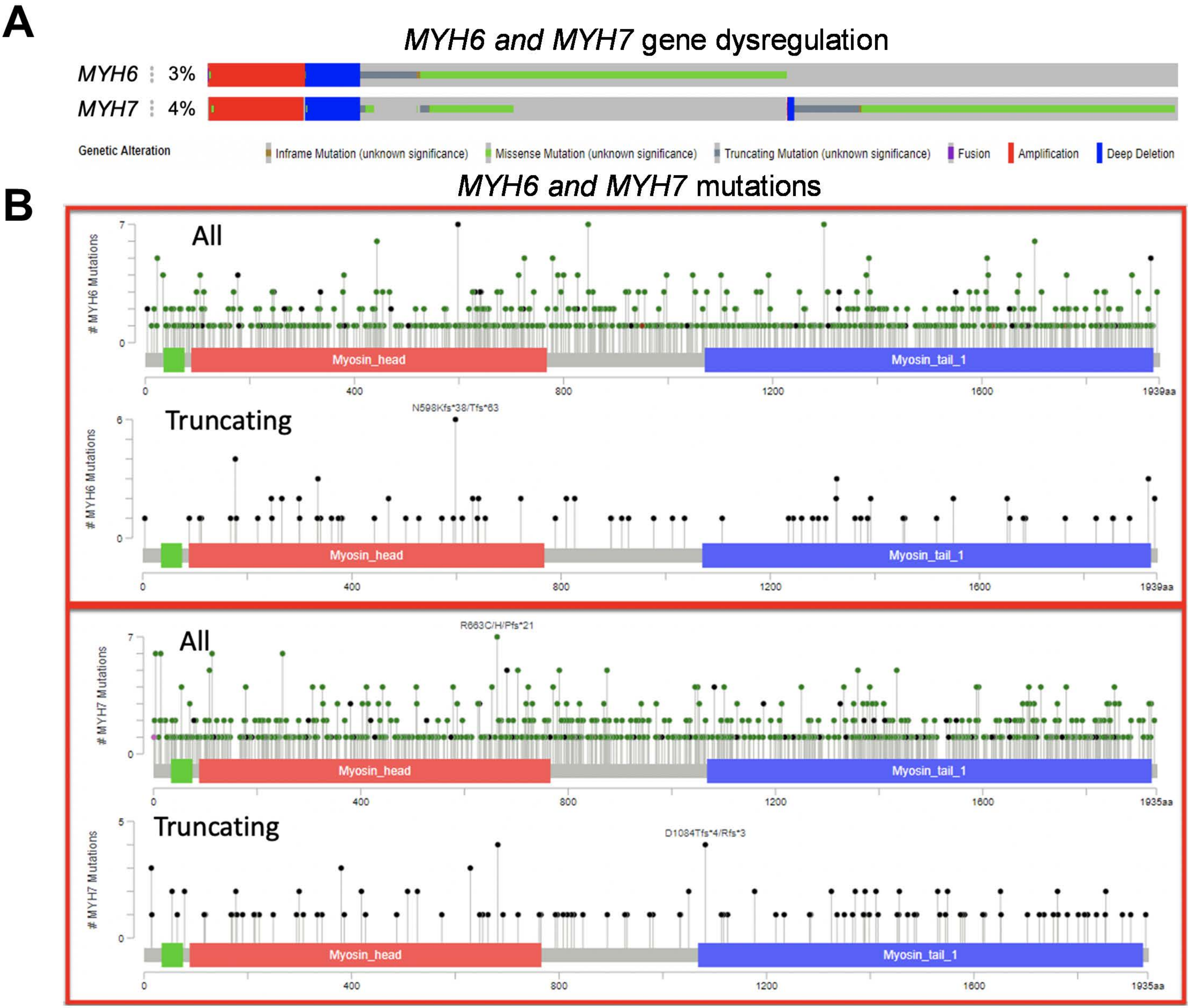

Supplemental Figure 3. Genomic dysregulation of cardiac myosin heavy chain MYH6 and MYH7 genes in cancer. **(A)** MYH6 and MYH7 genes are co-amplified or deleted in the same tumor samples (n=46697); **(B)** MYH6 and MYH7 genes are recurrently mutated in cancer (n=46697). To visualize mutations in these genes, we examined changes throughout the gene with the x-axis representing the amino acid number and the y-axis represents the total mutation counts at a specific residue. 960 mutations were observed in MYH6 while 1081 mutations for observed in MYH7. Missense (green) and truncation (black) mutations are shown. Truncation mutations are frequent and distributed throughout the two genes.

Supplemental Figure 3

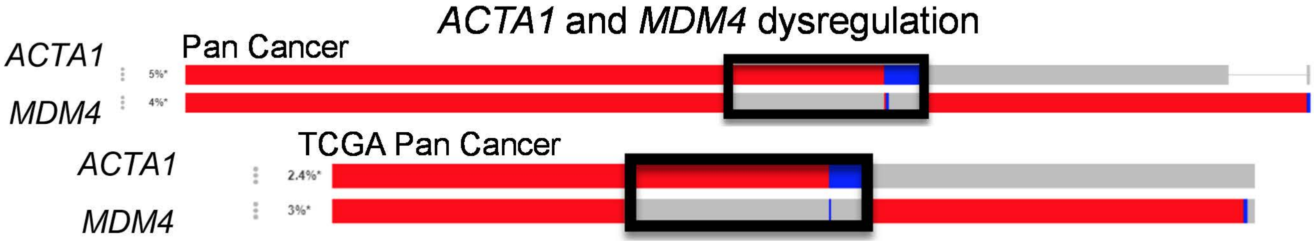

Supplemental Figure 4. Copy number variations of ACTA1 are observed in cancers (176 Pan Cancer studies). ACTA1, located next to MDM4 at 1q42.13, is amplified independent of MDM4 in limited patient tumor samples (black box).

Supplementary Figure 4

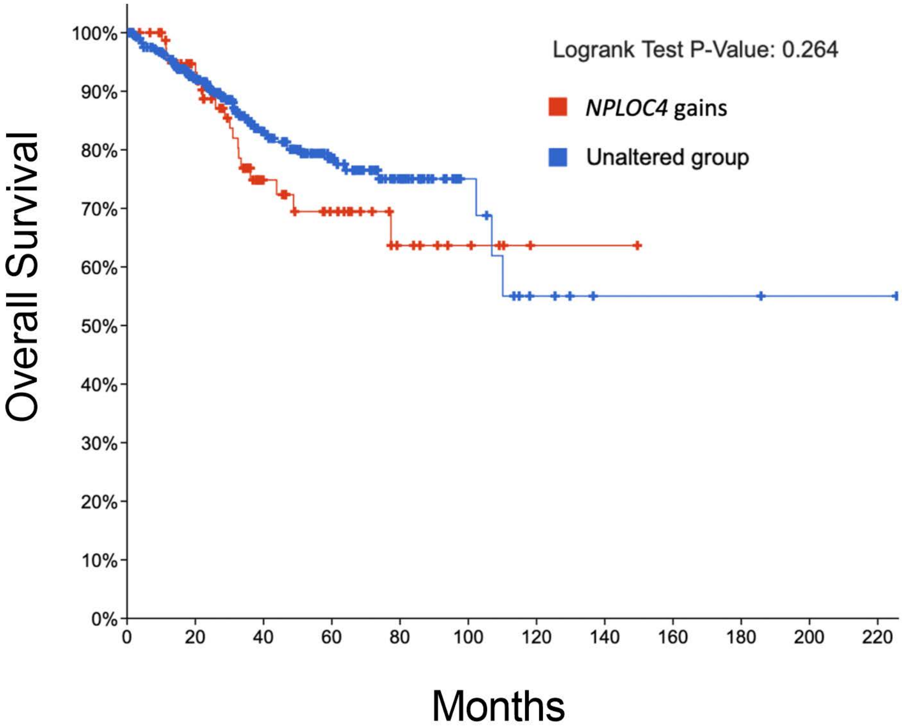

Supplemental Figure 5. Kaplan-Meier curve for NPLOC4 in UCEC. The altered group represents overall gain, including amplification and overexpression.

Supplementary Figure 5

Supplementary Table 1. Genomic location of nine myosin and actin genes of interest.

Gene	*MYL2*	*MYL3*	*MYH7*	*MYH6*	*MYLK2*	*MYLK3*	*ACTC1*	*ACTA1*	*ACTG1*
Name	Myosin Light Chain 2	Myosin Light Chain 3	Myosin Heavy Chain 7	Myosin Heavy Chain 6	Myosin Light Chain Kinase 2	Myosin Light Chain Kinase 3	Actin Alpha 1 Cardiac Muscle	Actin Alpha 1, Skeletal Muscle	Actin Gamma 1 Cytoplasmic
Genomic Location	12q24.11	3p21.31	14q11.2	14q11.2	20q11.21	16q11.2	15q14	1q42.13	17q25.3

Supplementary Table 2. Hallmark gene set ranking based on GSEA analysis of *ACTG1* gains in UCEC. Pathways are ranked by NES (Net Enrichment Score).

UCEC		
**Hallmark Gene Set**	**NES**	**FDR**
Interferon Gamma Response	-2.033	0
KRAS Signaling (Down)	2.14	0
Apical Junction	2.214	0
Myogenesis	2.2559	0
Allograft Rejection	-1.9148	0.0009
MYC Targets (V2)	1.8584	0.0009
Interferon Alpha Response	-1.8716	0.001
Epithelial Mesenchymal Transition	1.8687	0.0011
Protein Secretion	-1.829	0.0015
Cholesterol Homeostasis	1.8063	0.0018
Androgen Response	-1.6895	0.0065
Apical Surface	1.6405	0.0075
UV Response (UP)	1.561	0.0142
Hypoxia	1.5618	0.016
Estrogen Response (Late)	1.5198	0.019
IL6 JAK STAT3 Signaling	-1.4886	0.0402
KRAS Signaling (Up)	-1.429	0.0611
Coagulation	1.3776	0.0614
Glycolysis	1.3462	0.0743
Fatty Acid Metabolism	-1.3571	0.0765
Pancreas Beta Cells	-1.3633	0.0795
Complement	-1.3689	0.0841
Inflammatory Response	-1.3804	0.0851

Supplementary Table 3. Hallmark gene set ranking based on GSEA analysis of *ACTG1* gains in UCS. Pathways are ranked by NES (Net Enrichment Score).

UCS		
**Hallmark Gene Set**	**NES**	**FDR**
Interferon Gamma Response	-2.5541	0
Allograft Rejection	-2.3693	0
Interferon Alpha Response	-2.3287	0
Myogenesis	-2.268	0
IL6 JAK STAT3 Signaling	-2.2616	0
Inflammatory Response	-2.1501	0
Complement	-1.9128	0.0001
IL2 Stat5 Signaling	-1.7256	0.003
Xenobiotic Metabolism	-1.597	0.0102
Apical Surface	-1.5643	0.0144
Epithelial Mesenchymal Transition	-1.4791	0.0348
KRAS Signaling (Up)	-1.4572	0.0364
Adipogenesis	-1.4587	0.0385
Apoptosis	-1.444	0.0388
TNFA Signaling (Via NFKB)	-1.4121	0.0489
Estrogen Response Early	-1.3866	0.0606
Pancreas Beta Cells	-1.3791	0.0612

Supplementary Table 4. Oncogenic signaling program rankings based on GSEA analysis of *ACTG1* gains in UCEC and UCS.

Oncogenic Signaling Programs. C6	UCS-NES	UCS-FDR	UCEC-NES	UCEC-FDR
RPS14_DN.V1_UP	-1.993	0.000	-1.851	0.000
LTE2_UP.V1_DN	-1.754	0.006	-1.884	0.000
BMI1_DN.V1_UP	-1.713	0.009	1.923	0.001
HOXA9_DN.V1_UP	-1.661	0.011	-2.127	0.000
MEK_UP.V1_DN	-1.615	0.015	-2.278	0.000
STK33_SKM_UP	-1.579	0.015	-2.329	0.000
LEF1_UP.V1_UP	-1.588	0.014	1.893	0.001
LEF1_UP.V1_DN	-1.592	0.017	-1.862	0.000
MEL18_DN.V1_UP	-1.646	0.013	1.447	0.030
KRAS.50_UP.V1_DN	1.558	0.049	2.103	0.000
ATF2_UP.V1_UP	-1.452	0.049	1.926	0.001
AKT_UP.V1_DN	-1.569	0.015	1.401	0.041
EIF4E_DN	-1.427	0.062	-1.762	0.001
GCNP_SHH_UP_LATE.V1_DN	-1.390	0.077	2.021	0.000
KRAS.BREAST_UP.V1_DN	-1.419	0.062	1.547	0.018
KRAS.LUNG.BREAST_UP.V1_DN	1.357	0.083	1.800	0.003
JAK2_DN.V1_DN	1.364	0.089	-1.879	0.000
PRC1_BMI_UP.V1_DN	1.560	0.097	1.857	0.001
BCAT_GDS748_UP	-1.590	0.015	-1.393	0.088
KRAS.BREAST_UP.V1_UP	-1.372	0.082	1.512	0.022
ESC_J1_UP_LATE.V1_UP	-1.680	0.013	1.282	0.099
KRAS.PROSTATE_UP.V1_DN	1.369	0.100	1.412	0.040
CORDENONSI_YAP_CONSERVED_SIGNATURE	-1.401	0.074	1.295	0.093

Supplementary Table 5. Categorization of *ACTG1* dependent and non-dependent cell lines in uterine cancer.

Dependent Cell Lines		
**Cell Line**	**Lineage Subtype**	**Primary/Metastasis**
KLE	Endometrial Adenocarcinoma	Primary
HEC6	Endometrial Adenocarcinoma	Primary
EN	Endometrial Adenocarcinoma	Primary
ESS1	Endometrial Stromal Sarcoma	Primary
SNGM	Endometrial Adenocarcinoma	Metastasis
HEC50B	Endometrial Adenocarcinoma	Metastasis
HEC251	Endometrial Adenocarcinoma	Primary
JAR	Choriocarcinoma	Primary
MFE319	Endometrial Adenocarcinoma	N/A
SNU685	Carcinosarcoma	Primary
JHUEM7	Endometrial Adenocarcinoma	Primary

Non-Dependent Cell Lines		
**Cell Line**	**Lineage Subtype**	**Primary/Metastasis**
TEN	Clear Cell Carcinoma	Primary
HEC59	Endometrial Adenocarcinoma	Primary
HEC1B	Endometrial Adenocarcinoma	Primary
JHUEM1	Endometrial Adenocarcinoma	Primary
SNU1077	Carcinosarcoma	Primary
RL952	Endometrial Adenosquamous	Primary
HEC265	Endometrial Adenocarcinoma	Primary
SKGI	Endometrial Squamous	Primary
